# Novel therapeutic use of nemolizumab for refractory chronic scalp dysesthesia

**DOI:** 10.1016/j.jdcr.2025.12.014

**Published:** 2025-12-15

**Authors:** Lauren Kuan, Theodore Mah, Peter B. Chansky

**Affiliations:** a Schweiger Dermatology Group, San Francisco, California; b University of Pennsylvania, Philadelphia, Pennsylvania; c UCSF Health St. Mary’s Hospital, San Francisco, California

**Keywords:** nemluvio, nemolizumab, scalp dysesthesia, scalp itch, scalp pruritus

## Introduction

Scalp dysesthesia (SD), also known as burning scalp syndrome, is characterized by a generalized sensation of burning pain, itch, numbness, or tingling of the scalp without any primary skin lesions.^1^ Common symptomatic cutaneous dermatoses of the scalp, such as seborrheic dermatitis, lichen planopilaris, allergic or irritant contact dermatitis, folliculitis, and discoid lupus, must be treated and excluded before establishing a diagnosis of SD. Symptoms of SD are generally associated with physical or psychological stress. SD predominantly occurs in middle-aged women and is associated with underlying depression and anxiety.^2^^,^^3^ Chronic SD is also associated with cervical spine disease, stroke, multiple sclerosis, and trigeminal trophic syndrome. Treatment of SD is notoriously challenging, and therapeutic options are limited. As with the treatment of other dysesthesias, SD can be treated with either neurogenic or psychogenic medication.^4^^,^^5^ Current off-label treatments for SD include low-dose selective serotonin reuptake inhibitors, tricyclic antidepressants, anticonvulsants, low-dose naltrexone, gabapentin, pregabalin, botulinum toxin, topical corticosteroids, topical anesthetics, topical ketamine, topical amitriptyline, and capsaicin cream.^2^ Nemolizumab, an interleukin (IL) 31 receptor ɑ antagonist approved by the US Food and Drug Administration for atopic dermatitis and prurigo nodularis, inhibits IL-31 signaling and improves symptoms of pruritus; however, nemolizumab has not been studied in the treatment of SD.^6^, ^7^, ^8^, ^9^, ^10^ Herein, we present a novel case of chronic SD treated successfully with nemolizumab.

## Case presentation

A 41-year-old woman presented to our clinic with chronic pruritus of the scalp for 3 years. Her past medical history was significant for asthma and a past diagnosis of anxiety for which she was on selective serotonin reuptake inhibitor therapy from 2010 to 2011. She was previously evaluated by a dermatologist and prescribed clobetasol 0.05% ointment and calcipotriene 0.005% ointment for presumed scalp psoriasis. She reported that clobetasol improved but did not cure her itch symptoms, while calcipotriene and over-the-counter antidandruff products worsened them. On physical exam, only mild seborrheic dermatitis was present. The patient was subsequently empirically treated for seborrheic dermatitis and prescribed ketoconazole 2% shampoo (3 times weekly) for maintenance and clobetasol 0.05% ointment (twice daily for 2 weeks) as needed for flares. The patient was not on any oral medications at the time.

At her 6-week follow-up visit, there was no active seborrheic dermatitis, scale, or inflammation of the scalp; however, she continued to experience significant scalp pruritus, with a Peak Pruritus Numeric Rating Scale (PP-NRS) score of 7 without topical steroid intervention (scale: 0-10) and an Investigator’s Global Assessment score of 3 (scale: 0-4). A diagnosis of chronic SD was established.

Due to lack of efficacy with standard treatments and her predominant symptom of pruritus, the patient was offered treatment with a selective serotonin reuptake inhibitor or a tricyclic antidepressant. The patient declined these therapies due to the side-effect profile and inconvenience of taking a daily pill. Thus, nemolizumab was considered as an off-label treatment option. The patient was subsequently initiated on nemolizumab with a loading dose of 60 mg injected subcutaneously, followed by a dose of 30 mg every 4 weeks. Within 5 days after the initial dose, the patient noted a significant improvement in her symptom of scalp pruritus. After 1 month of treatment, her SD had fully resolved, and she reported a PP-NRS itch score of 0 without use of topical corticosteroids or ketoconazole 2% shampoo. She noted that between her monthly injections, her most severe itching reached a PP-NRS score of 2, for which she applied clobetasol 0.05% ointment during exacerbations. After 13 weeks of therapy, her PP-NRS itch score has remained 0 and her scalp has remained clear with no evidence of scale or inflammation—indicating a sustained response on nemolizumab 30 mg every 4 weeks. She has experienced no side effects while on nemolizumab.

## Discussion

To our knowledge, this is the first reported case worldwide of successful treatment of chronic SD with nemolizumab. Chronic SD with pruritus is extremely challenging to manage with no current Food and Drug Administration-approved therapies. All current treatment options are based on small case series, and no one therapy is effective for every patient. Furthermore, there are limited options to guide treatment once the first and second line options fail.

Nemolizumab has an uncommon mechanism of action and is the only neuroimmune treatment on the market that competitively blocks the IL-31 cytokine and its downstream signaling pathways. IL-31 plays a key role in itch and is upregulated in the skin of patients with atopic dermatitis and prurigo nodularis. Patients with SD often complain of significant pruritus and possibly have elevated levels of IL-31 in their skin. Thus, by blocking the effect of IL-31 and improving itch and inflammation, nemolizumab has therapeutic potential in the treatment of SD and SD-associated pruritus. The nemolizumab dosing guide for atopic dermatitis is 60 mg at day 0 and then 30 mg every 4 weeks, with the possibility of tapering to every 8 weeks in patients who are clear or almost clear. Given this, we could consider tapering our patient to nemolizumab 30 mg every 8 weeks in the future.

In conclusion, this case highlights the promising potential of nemolizumab as a fast, effective, and safe treatment option for chronic refractory SD. Further research is necessary to assess the successful applicability of this novel therapy in larger cohorts of patients with SD.

## Conflicts of interest

Dr Chansky is a paid speaker for Sun Pharmaceuticals, Inc, and Sanofi and serves on the advisory board for Regeneron Pharmaceuticals, Inc.
